# Gamma Knife Radiosurgery for Acromegaly

**DOI:** 10.1155/2012/821579

**Published:** 2012-02-13

**Authors:** John D. Rolston, Lewis S. Blevins

**Affiliations:** ^1^Department of Neurological Surgery, University of California, San Francisco, San Francisco, CA 94143-0112, USA; ^2^California Center for Pituitary Disorders, University of California, San Francisco, San Francisco, CA 94143-0350, USA

## Abstract

Acromegaly is debilitating disease occasionally refractory to surgical and medical treatment. Stereotactic radiosurgery, and in particular Gamma Knife surgery (GKS), has proven to be an effective noninvasive adjunct to traditional treatments, leading to disease remission in a substantial proportion of patients. Such remission holds the promise of eliminating the need for expensive medications, along with side effects, as well as sparing patients the damaging sequelae of uncontrolled acromegaly. Numerous studies of radiosurgical treatments for acromegaly have been carried out. These illustrate an overall remission rate over 40%. Morbidity from radiosurgery is infrequent but can include cranial nerve palsies and hypopituitarism. Overall, stereotactic radiosurgery is a promising therapy for patients with acromegaly and deserves further study to refine its role in the treatment of affected patients.

## 1. Introduction

Acromegaly is serious endocrinological derangement which, left untreated, reduces life expectancy and results in physiological derangements and complications that may negatively affect a patient's quality of life. The first line of treatment is typically surgical resection of the tumor causing acromegaly. Those patients with residual or recurrent disease are often treated with medication to decrease growth hormone secretion or block its action on peripheral tissues. These treatments are not universally effective for patients and are sometimes contraindicated. As an adjunct treatment, and sometimes as an alternative treatment, radiosurgery has proven to be an attractive therapy. It is noninvasive, has few side effects, and is available in many centers internationally [1–3]. In the current paper, we first describe the pathophysiology of acromegaly, the existing surgical and medical treatments, and then introduce radiosurgical methods. In particular, we will focus on gamma knife radiosurgery (GKS) since it has a broader base of supporting literature than alternative forms of stereotactic radiosurgery. Finally, the overall efficacy of GKS is described, along with its reported morbidities.

## 2. Acromegaly

Acromegaly is a syndrome caused by elevated levels of circulating growth hormone (GH). The most common cause of the disorder, accounting for about 98% of cases, is a GH-secreting pituitary adenoma. Rare nonpituitary causes of acromegaly include diverse entities such as hypothalamic hamartomas, small-cell lung cancers, pheochromocytomas, and bronchial carcinoids. Among GH-secreting pituitary adenomas, roughly 60% are pure GH-secreting somatotrope adenomas, while the remainder are mixed mammosomatotropes, which secrete both GH and prolactin (PRL) and sometimes thyroid-stimulating hormone (TSH) [4].

The symptoms of increased GH are mediated by both the direct effects of GH binding to the GH receptor, activating the JAK/STAT pathway, and indirectly via insulin-like growth factor 1 (IGF-1). IGF-1 is synthesized predominantly in the liver under the control of GH. The combined effects of GH and IGF-1 on target tissues lead to bony and soft tissue growth, noted by a characteristic constellation of signs including frontal bossing, prognathism, widely spaced teeth (due to mandibular growth), increased shoe or ring sizes, skin tags, carpal tunnel syndrome, and coarse facial features [4].

These externally recognizable signs in themselves are not as important to overall morbidity as internal changes, including cardiomegaly and visceromegaly [4, 5]. Diabetes mellitus occurs in roughly 25% of patients, and cardiomyopathy with arrhythmia, hypertension, and diastolic dysfunction occurs in 30%. Additionally, colonic polyps are more frequent, as is sleep apnea which occurs in 60% of patients, presumably secondary to soft tissue expansion and macroglossia. When uncontrolled, acromegaly reduces life expectancy by 10 years [4]. 

The prevalence of acromegaly is uncertain. Multiple studies through the 1920s to 1990s agreed on a prevalence of roughly 60 per million, a mean age of onset of 44 years, symptoms lasting on average 8 years before diagnosis, and no difference in incidence between men and women [6]. However, more recent studies place the prevalence at 86 per million [7], 124 per million [8], and a remarkably high 1034 per million [9]. However, in addition to being geographically and demographically restricted (primary care patients in Germany), this last study was based on a biochemical definition of acromegaly (elevated IGF-1 and GH) rather than a syndrome definition, and therefore will include many patients who would otherwise not seek treatment [9]. Such a more liberal definition might be one method of discovering at-risk patients earlier in the course of their disease and permitting more time to mitigate the accruing morbidity.

## 3. Conventional Treatment

Surgery is typically the first line of treatment for acromegaly due to pituitary adenomas provided there are no surgical contraindications. Residual disease is then managed medically with somatostatin analogues, dopamine agonists, or GH receptor antagonists [10].

### 3.1. Surgical

Most adenomas are resected via the transsphenoidal approach (transsphenoidal adenomectomy, TSA). Using either a microscope or endoscope, the surgeon enters the sphenoid sinus transnasally, then penetrates the sella, and finally debulks and removes the tumor, sparing as much of the normal pituitary as possible. For microadenomas (<10 mm in diameter), TSAs lead to correction of GH levels in ~70% of patients [11]. Only 50% of patients with macroadenomas achieve normalization [11].

### 3.2. Medical

For those patients who do not experience normalization of GH levels after surgery, or who are not surgical candidates, medical therapy is initiated. The first drug is usually a somatostatin analogue such as octreotide or lanreotide. The LAR (long-acting release) formulation of octreotide uses polymeric microspheres and is injected monthly [12]. Lanreotide ATG (autogel) is the only formulation of lanreotide currently available in the United States and is suspended in aqueous solution in microsyringes for subcutaneous delivery by the patient. The two formulations appear to be equally efficacious and normalize IGF-I levels in 50–60% of patients [13–16]. Somatostatin analogues appear to induce tumor shrinkage in approximately 42% of patients, when data are pooled across studies [17]. Interestingly, however, tumor shrinkage appears more pronounced in primarily treated patients (52%) as opposed to patients receiving adjunctive, postsurgical treatment (21%) [17].

Dopamine agonists appear to work best in patients whose tumors also secrete prolactin [18]. Among dopamine agonists, cabergoline appears to be the most efficacious, resulting in normalization of IGF-I levels in 34% of patients [19]. However, no dopamine agonist alone is as effective as a somatostatin analogue [20], although there might be synergistic effects when used in conjunction with somatostatin analogues [21–24].

Pegvisomant is a PEGylated growth hormone analogue that is subcutaneously injected by patients. In one study, treatment for 12 months led to normalized IGF-1 levels in 97% of patients [25], and treatment for 24 months led to normalization in 76.3% of patients in a different study [26]. However, the side effects of pegvisomant include transaminitis, lipodystrophy at injection sites, and, most worrisome, possible tumor progression, due to blocking of the normal inhibitory feedback of growth hormone levels of the adenoma [27–29].

## 4. Radiosurgery

### 4.1. Technology

Simply put, resective surgery is a means of removing unwanted tissue from the body. Radiosurgery (RS) achieves the identical goal but, rather than directly removing cells, induces cell death instead. This cell death can be induced either directly, via necrosis or apoptosis, or indirectly, by damaging the tissue blood supply. When inducing cell death, RS uses ionizing radiation, wherein charged particles or photons strip electrons from atoms and molecules, thereby damaging DNA, proteins, and other molecules within cells and in the extracellular matrix. When this damage cannot be repaired, cells undergo either apoptosis or necrosis [30, 31].

Various types of radiosurgery are available, but each works by emitting charged particles or photons. Proton beams, for example, generated by particle accelerators and can be used to target structures deep within the cranium. This is due, in part, to the characteristic peak and subsequent drop-off of radiation intensity in proton beams—the Bragg peak [31]. The depth of this peak can be modulated and is used to focus the effects of the radiation on particular structures, sparing healthy ones. The drawback to proton therapy, however, is the high cost and relative scarcity of facilities capable of providing therapeutic proton beams [32].

Photons, on the other hand, are much more commonly used for radiosurgery. The two major techniques are linear accelerators and radioactive isotopes. Linear accelerators, like the CyberKnife (Accuray Inc., Sunnyvale, CA), produce photons which are then aimed toward deep structures within the brain or spine. To limit damage along the path of the beam, a weak beam is used and moved around the patient. Thus, the target of the beam is constant, always receiving radiation, but the intervening structures are exposed only briefly [30–32].

The Gamma Knife (Elekta AB, Stockholm, Sweden), developed by Lars Leksell in 1968, uses 201 sources of radioactive cobalt-60 [32]. Each source generates a beam of photons as the cobalt decays, and these beams are focused on a central target within the brain through the use of collimators. Because each individual beam is weak, intervening tissue is exposed to far less radiation than the central target, which is the common focus of all 201 beams. The Gamma Knife has been around longer than most linear accelerator radiosurgical devices and is therefore a better-studied tool for use by neurosurgeons, though direct comparisons between the Gamma Knife and linear accelerators will undoubtedly change the prevailing practices in the future. The remainder of this paper will focus exclusively on GKS.

### 4.2. Clinical Studies

A large number of small case series have been carried out to evaluate the effects of Gamma Knife surgery (GKS) on acromegaly (Table 1). Most use remission criterion of a normal IGF-1 level and many add the criterion GH < 1 to 2 ng/mL, but it should be noted that remission criteria vary across studies. These criteria are roughly in line with the criteria set forth by the Acromegaly Consensus Group on 2010 (normal IGF-1 and GH < 1 ng/mL) [33]. Also variable between studies is the follow-up time, along with radiation dose, targeting protocol, and, most critically, pre-GKS therapy. This last note is particularly important since most series include patients who have already received transsphenoidal surgical resection as an inadequate treatment. Unfortunately, the data are not presented in the reviewed papers in such a way as to separate out response rates in patients receiving prior treatment versus those who were treated primarily with GKS.

Overall, however, the results from these studies suggest that GKS is an effective treatment for acromegaly. Across the 29 studies and 964 cases examined, 43% of patients achieved remission. The time to remission is not reliably reported, but most studies agree that the further out from RS patients are examined, the more likely they are to achieve a cure. This effect, intriguingly, does not seem to have a ceiling. For example, Vik-Mo et al. [34] showed an increase from 58% of patients with normal IGH-1 levels to 86% of patients over the time span of 5 to 10 years after RS. How exactly the effects of RS continue to evolve over such a protracted time is unknown, but consistent with how RS affects other diseases, like epilepsy [35] and vestibular schwannoma [36].

Interestingly, there is some (still debated) evidence that the use of antiacromegalic medicines prior to irradiation attenuates the effects of GKS [1]. That is, using somatostatin analogues or GH receptor antagonists has been shown in select studies to decrease a patient's chances of remission [37–39]. Though this has not been thoroughly examined, the mechanism is believed to be suppression of tumor cell cycle, thereby making the cells less prone to radiation-induced damage.

**Figure 1:**
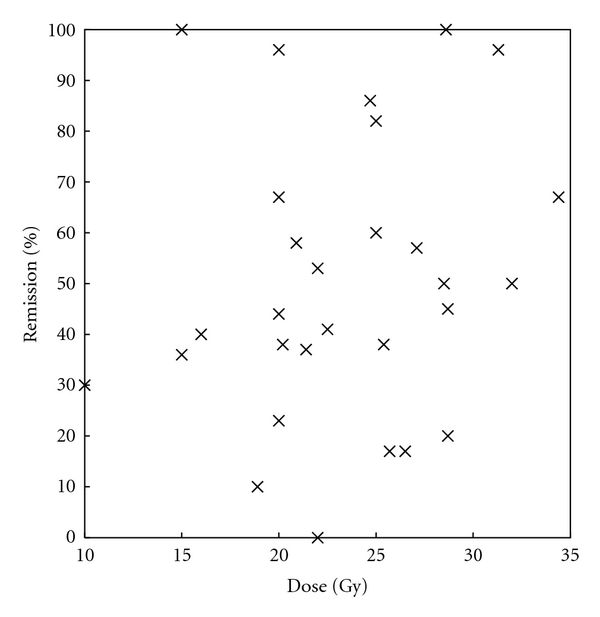
Effect of GKS dose on remission. The percentage of patients in remission is plotted as a function of the dose used during GKS. There is no significant trend, though the heterogeneity of the studies makes it difficult to draw definite conclusions.

There does not appear to be a correlation between radiation dose and rates of remission (Figure 1). However, the heterogeneity in study design, follow-up, and definition of remission makes such conclusions fraught.

### 4.3. Morbidity

**Figure 2:**
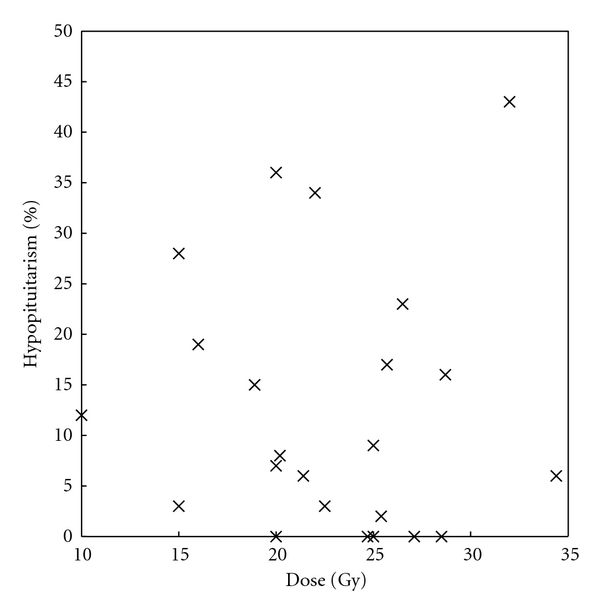
Effect of GKS dose on hypopituitarism as an adverse event. The rate of hypopituitarism is shown as a function of GKS dose for each study. There is no definite relationship. Again, however, the heterogeneity in study design, follow-up, and other parameters makes it difficult to draw definitive conclusions from this summary.

The most common complication of GKS for acromegaly is hypopituitarism, presumably from damage to the normal gland during irradiation, ranging from 0 to 43% [1] (Table 2). However, the degree to which such damage is a pure result of GKS versus prior surgery, if done, is unclear [1]. Moreover, there is no discernible relationship between radiation dose and the incidence of this complication (Figure 2), though these are very heterogeneous studies and it is possible that better-controlled or larger studies would uncover such relationships if they exist.

Other complications are inadequately documented but include headache, epilepsy, carotid artery stenosis, and, more frequently, cranial nerve palsies or neuropathies (including trigeminal neuralgia and visual decline) [1] (Table 2). The rate of visual disturbances seems to be, in the worst case, 11% [40, 41], though most trials either do not report these adverse events or show them to be on the order of 0–6% (Table 2).

Nevertheless, these complications should be viewed in light of the complications inherent to uncontrolled acromegaly or, if being used as an alternative to surgery, the morbidity of an endonasal neurosurgical procedure.

## 5. Conclusions

Acromegaly is a serious disorder leading to increased morbidity and mortality in patients. While surgical resection remains the first line of treatment, stereotactic radiosurgery is proving itself a feasible alternative therapy (when surgery might be contraindicated) and adjunct (when surgical resection is unsuccessful in leading to complete disease remission). GKS is very effective, leading to remission in over 40% of cases. But it is clearly not universally effective. Ultimately, further studies are needed to delineate which patients are most likely to receive benefit from GKS, along with ways to improve GKS outcomes.

## Figures and Tables

**Table 1:** Studies of GKS for acromegaly.

Study	No. of patients	Mean follow-up (mos.)	Dose (Gy)	No. of remission (%)
Iwai et al., 2010 [42]	26	84	20.2	10 (38)
Ronchi et al., 2009 [43]	28	103	20	19 (96)
Swords et al., 2009 [44]	10	38.5	10	3 (30)
Wan et al., 2009 [45]	103	67	21.4	38 (37)
Jagannathan et al., 2009 [46]	95	57	22	50 (53)
Losa et al., 2008 [47]	83	69	25	50 (60)
Pollock et al., 2008 [48]	27	48	20	18 (67)
Vik-Mo et al., 2007 [34]	53	66	26.5	9 (17)
Ježková et al., 2006 [49]	96	54	32	48 (50)
Castinetti et al., 2005 [50]	82	49.5	25.7	14 (17)
Kobayashi et al., 2005 [40]	67	63	18.9	7 (10)
Attanasio et al., 2003 [51]	30	46	20	7 (23)
Choi et al., 2003 [52]	12	42.5	28.5	6 (50)
Jane Jr. et al., 2003 [53]	64	>18	15	23 (36)
Petrovich et al., 2003 [54]	6	41	15	6 (100)
Ikeda et al., 2001 [55]	17	56	25	14 (82)
Fukuoka et al., 2001 [56]	9	42	20	4 (44)
Izawa et al., 2000 [57]	29	>6	22.5	12 (41)
Shin et al., 2000 [58]	6	43	34.4	4 (67)
Zhang et al., 2000 [59]	26	36	31.3	25 (96)
Inoue et al., 1999 [60]	12	>24	20.9	7 (58)
Kim et al., 1999 [61]	2	12	22	0 (0)
Kim et al., 1999 [62]	11	27	28.7	5 (45)
Mokry et al., 1999 [63]	10	46	16	4 (40)
Lim et al., 1998 [64]	16	25.5	25.4	6 (38)
Martinez et al., 1998 [65]	7	36	24.7	6 (86)
Morange-Ramosa et al., 1998 [66]	15	20	28.7	3 (20)
Pan et al., 1998 [67]	15	29	28.6	15 (100)
Park et al., 1996 [68]	7	15	27.1	4 (57)

Total	964			417 (43)

**Table 2:** Adverse events of GKS for acromegaly.

Study	Hypopituitarism (%)	Headache (%)	Radiation necrosis (%)	Visual changes (%)
Iwai et al., 2010 [42]	2 (8)	1 (4)	1 (4)	0
Ronchi et al., 2009 [43]	—	—	—	0
Swords et al., 2009 [44]	3 (12)	0		0
Wan et al., 2009 [45]	6 (6)	—	2 (2)	0
Jagannathan et al., 2009 [46]	32 (34)	—	—	4 (4)
Losa et al., 2008 [47]	7 (9)	5 (6)	—	0
Pollock et al., 2008 [48]	16 (36)	—	—	1 (2)
Vik-Mo et al., 2007 [34]	14 (23)	—	—	—
Ježková et al., 2006 [49]	26 (43)	—	—	—
Castinetti et al., 2005 [50]	14 (17)	—	—	1 (1)
Kobayashi et al., 2005 [40]	39 (15)*	—	—	29 (11)*
Attanasio et al., 2003 [51]	2 (7)	1 (3)	—	0
Choi et al., 2003 [52]	0	—	—	0
Jane Jr. et al., 2003 [53]	18 (28)	—	—	—
Petrovich et al., 2003 [54]	2 (3)*	2 (3)*	—	3 (4)*
Ikeda et al., 2001 [55]	0	—	—	0
Fukuoka et al., 2001 [56]	0	—	—	0
Izawa et al., 2000 [57]	1 (3)	—	1 (3)	1 (3)
Shin et al., 2000 [58]	1 (6)*	—	—	1 (6)*
Zhang et al., 2000 [59]	—	—	—	—
Inoue et al., 1999 [60]	—	—	—	—
Kim et al., 1999 [61]	—	—	—	—
Kim et al., 1999 [62]	—	—	0	0
Mokry et al., 1999 [63]	3 (19)	—	—	0
Lim et al., 1998 [64]	1 (2)*	18 (28)*	—	1 (2)*
Martinez et al., 1998 [65]	0	—	—	1 (3)*
Morange-Ramosa et al., 1998 [66]	4 (16)*	—	—	1 (4)*
Pan et al., 1998 [67]	—	—	—	—
Park et al., 1996 [68]	0	—	0	0

Total (%)	191 (16)	27 (12)	4 (2)	43 (4)

*Numbers of GKS for all treated pituitary tumor types (not reported for acromegalic patients alone).
